# The giant resectable carcinoma of gall bladder—a case report

**DOI:** 10.1186/s12893-021-01117-2

**Published:** 2021-03-16

**Authors:** Lovenish Bains, Haraesh Maranna, Pawan Lal, Ronal Kori, Daljit Kaur, Varuna Mallya, Veerpal Singh

**Affiliations:** 1 Department of Surgery, Maulana Azad Medical College, New Delhi, India; 2 Department of Transfusion Medicine, All India Institute of Medical Sciences, Rishikesh, India; 3 Department of Pathology, Maulana Azad Medical College, New Delhi, India

**Keywords:** Giant, Gall bladder (GB), Carcinoma, Gall bladder cancer (GBC), Cholecystectomy

## Abstract

**Background:**

Gall bladder cancer (GBC) is the fifth most common malignancy in the gastrointestinal system and the most common malignancy of the biliary system. GBC is a very aggressive malignancy having a 5 year survival rate of 19%. Giant Gall Bladder (GGB) is an uncommon condition that can result from cholelithiasis or chronic cholecystitis and rarely with malignancy.

**Case report:**

A 65 year old lady presented with vague abdominal pain for 12 years and right abdominal lump of size 20 × 8 cms was found on examination. CT scan showed a circumferentially irregularly thickened wall (2.5 cm) of gall bladder indicative of malignancy. Per-operatively a GB of size 24 × 9 cm was noted and patient underwent radical cholecystectomy. It was surprise to find such a giant malignant GB with preserved planes. Histopathology, it was well differentiated adenocarcinoma of gall bladder of Stage II (T2a N0 M0).

**Discussion:**

It is known that mucocoele of GB can attain large size, however chronic cholecystitis will lead to a shrunken gall bladder rather than an enlarged one. A malignant GB of such size and resectable is rare without any lymph node involvement or liver infiltration. Few cases of giant benign gall bladder have been reported in literature, however this appears to be the largest resectable gall bladder carcinoma reported till date as per indexed literature.

**Conclusion:**

Giant GB is an uncommon finding. They are mostly benign, however malignant cases can occur. Radiological findings may suggest features of malignancy and define extent of disease. Prognosis depends on stage of disease and resectability, irrespective of size.

## Background

Gall Bladder Cancer (GBC) is the most common biliary tree malignancy worldwide accounting for 80–95% of malignancies and the fifth most common malignancy of gastro intestinal system [1]. The incidence varies globally with high incidences in Northern India, South America and Pakistan [2]. GBC is a very aggressive malignancy having a 5 year survival rate of 19% as per the Surveillance, Epidemiology, and End Results (SEER) database, hence a satisfactory outcome depends on early diagnosis of the disease and aggressive surgical resection [3]. Despite the possibility of cure in early disease, only 20% of patients have resectable tumors at presentation and about 50% of the cases have lymph nodal involvement [3, 4]. Giant gall bladder (GGB) is an uncommon condition that can result from cholelithiasis or chronic cholecystitis [5]. It is known that mucocoele of Gall Bladder (GB) can attain large size reaching up to iliac fossa, however a malignant GB of such size and resectable is rare without any lymph node involvement or liver infiltration. We report our experience of a giant carcinoma gall bladder of size 24 × 9 cms with albeit surprisingly no infiltration and preserved planes in a 65 year old lady who underwent radical cholecystectomy.

## Case Presentation

A 65 year old lady presented with a progressive swelling in right side of abdomen for 8 months. She had vague right sided abdominal discomfort and heaviness for the past 12 years. There was no history of jaundice, vomiting or abdominal distension. Her bowel and bladder habits were unremarkable. Her past medical history, menstrual history was also unremarkable. On examination she was anicteric, vitals stable and with a Karnofsky performance scale of 80. Her BMI was 21. There was fullness of right side abdomen on inspection and further examination revealed a non-tender, firm lump of approximately 20 × 8 cms extending from right hypochondrium to right iliac fossa, whose upper and lower margins were not palpable. There was slight side to side mobility. Liver was not enlarged, but the lump was contiguous with the liver, no nodules were palpable on the liver surface and no sign of ascites was found.

**Fig. 1:**
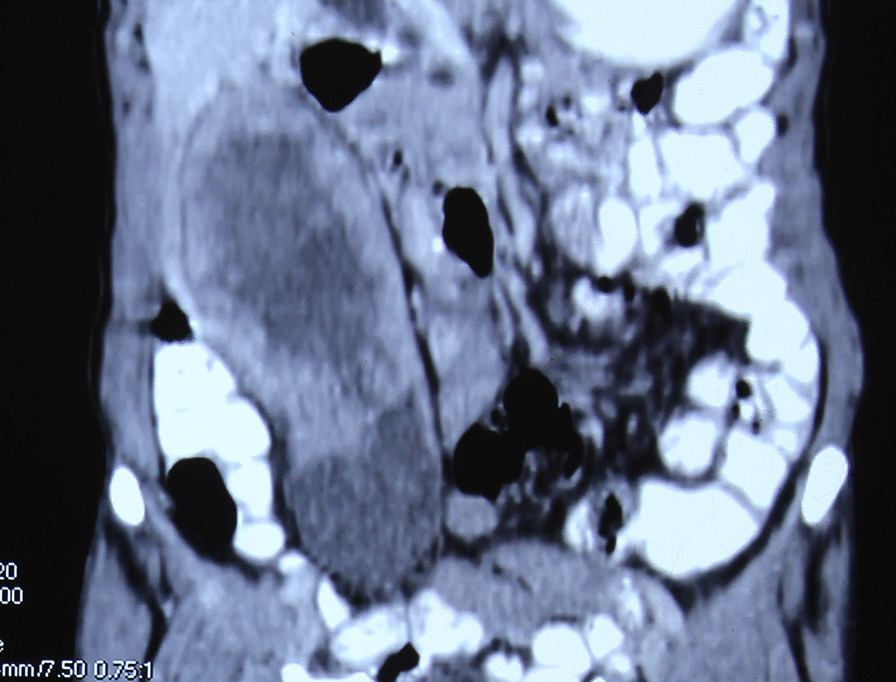
CT scan (coronal plane) showing large GB reaching towards pelvis

**Fig. 2:**
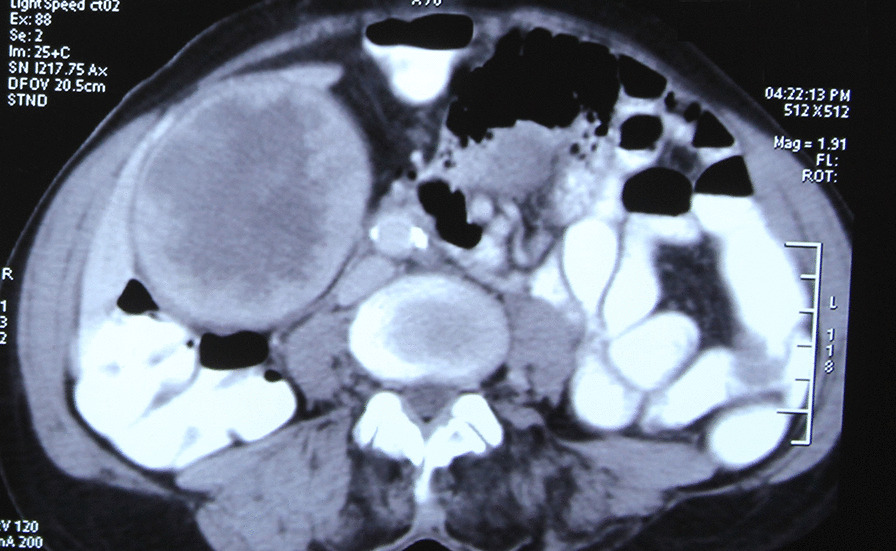
CT scan (transverse plane) showing thickened irregular GB wall

**Fig. 3:**
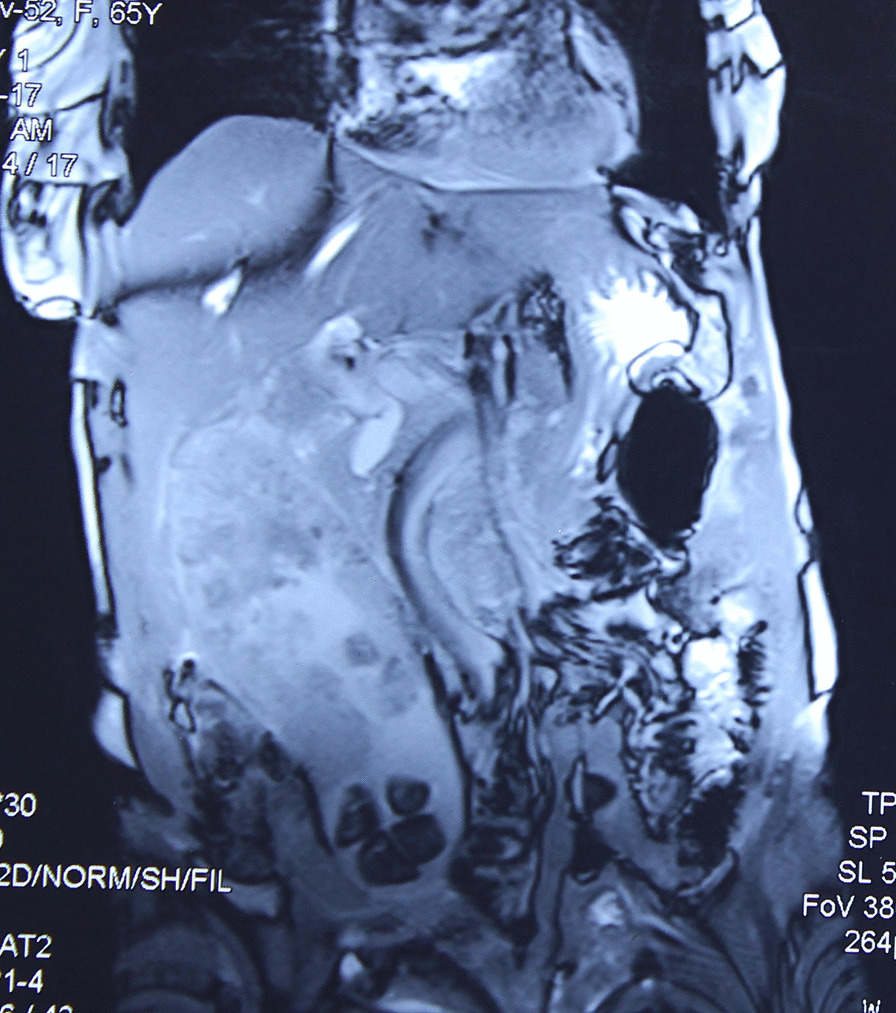
MRCP (T1 image) showing irregular thickened walls with large stones

**Fig. 4:**
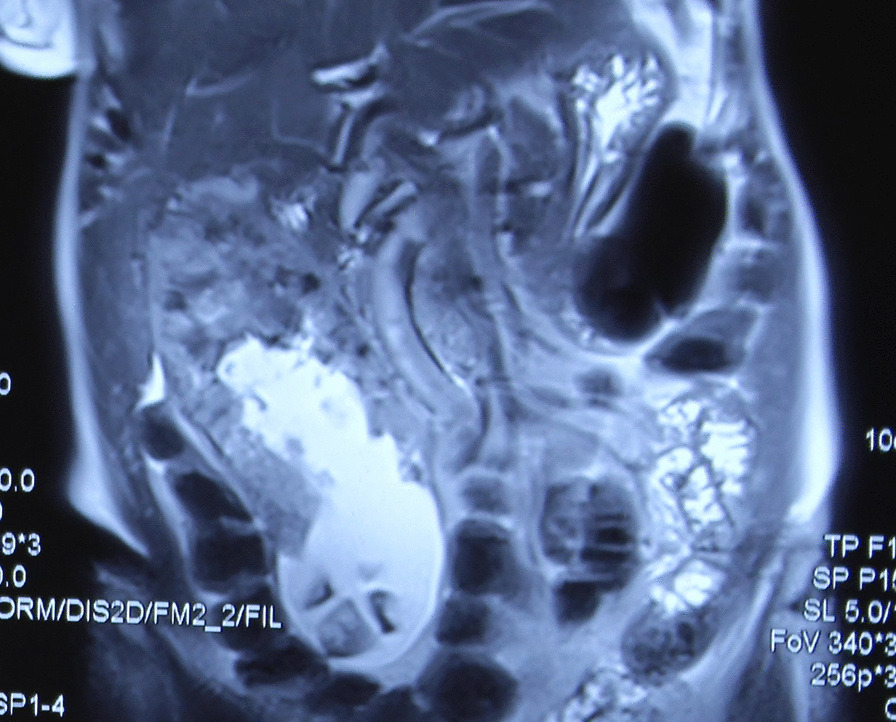
MRCP (T2 image) showing bulk of tumor in neck and body

Ultrasonogram of abdomen revealed a massively distended gall bladder with multiple gall stones, largest of size 4 cm and circumferentially irregularly thickened wall. Computerized Tomography of the abdomen (performed 2 months before in another institution) showed a hugely distended gall bladder of size 23 × 8 cm with markedly thickened walls with multiple neovascularization along the neck of gall bladder suggestive of neoplastic etiology. (Figs. 1, 2) There were maintained fat planes between the gall bladder with liver, duodenum, ascending colon, and portal vein. Liver was normal and there was no evidence of metastatic deposits. Magnetic resonance cholangiopancreatography at our institution revealed heterogeneously enhancing irregular polypoidal circumferential mural thickening noted in the region of body and neck of gall bladder measuring 2.5 cms in maximum thickness, appearing hypointense on T1 WI and hyperintense on T2 WI (Figs. 3, 4). Multiple calculi (average diameter—3.5 cm) were noted in the gall bladder lumen. Her laboratory parameters including liver function tests were within normal range. Serum Carbohydrate antigen 19-9 (CA 19.9) was 11 units/mL (normal 0–37 units/mL).

**Fig. 5:**
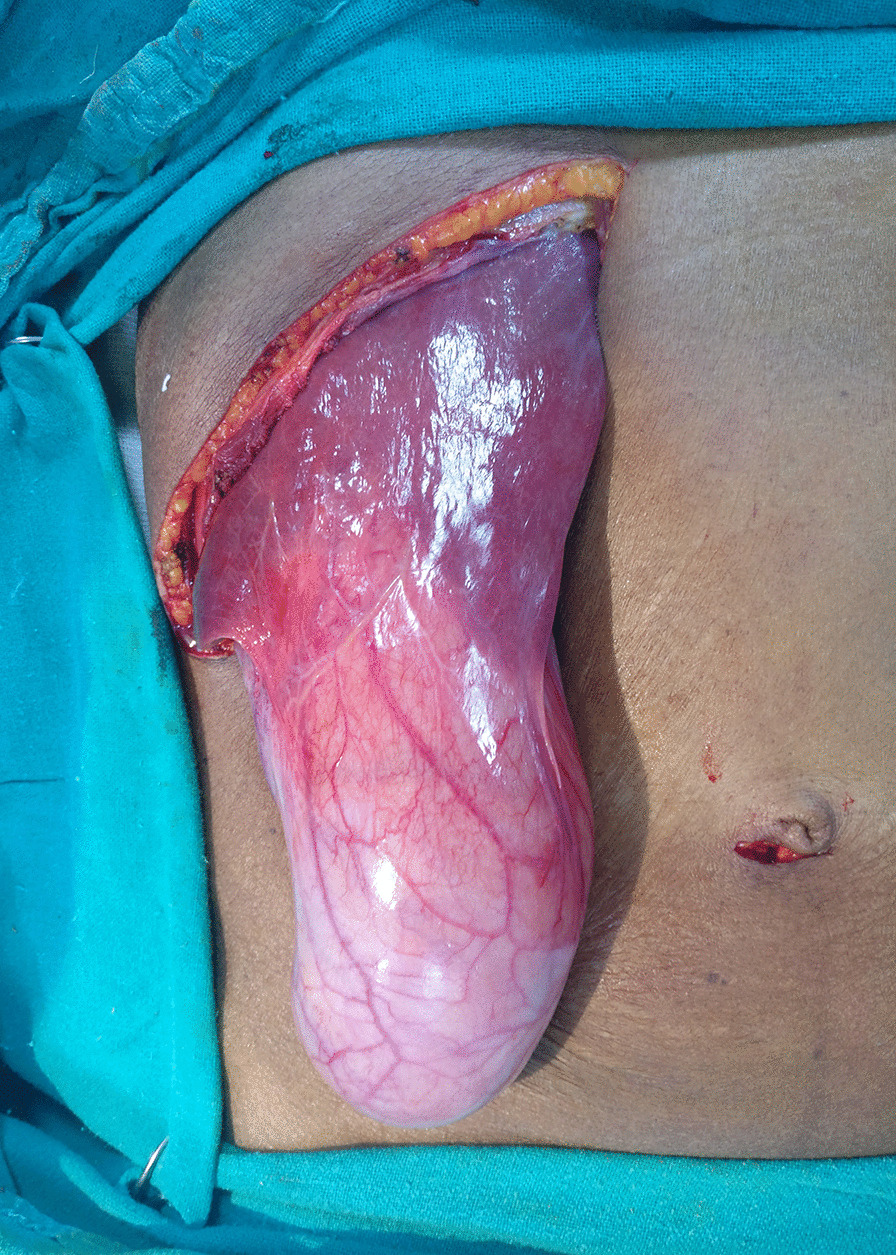
Intra-operative- GB till anterior superior iliac spine

**Fig. 6:**
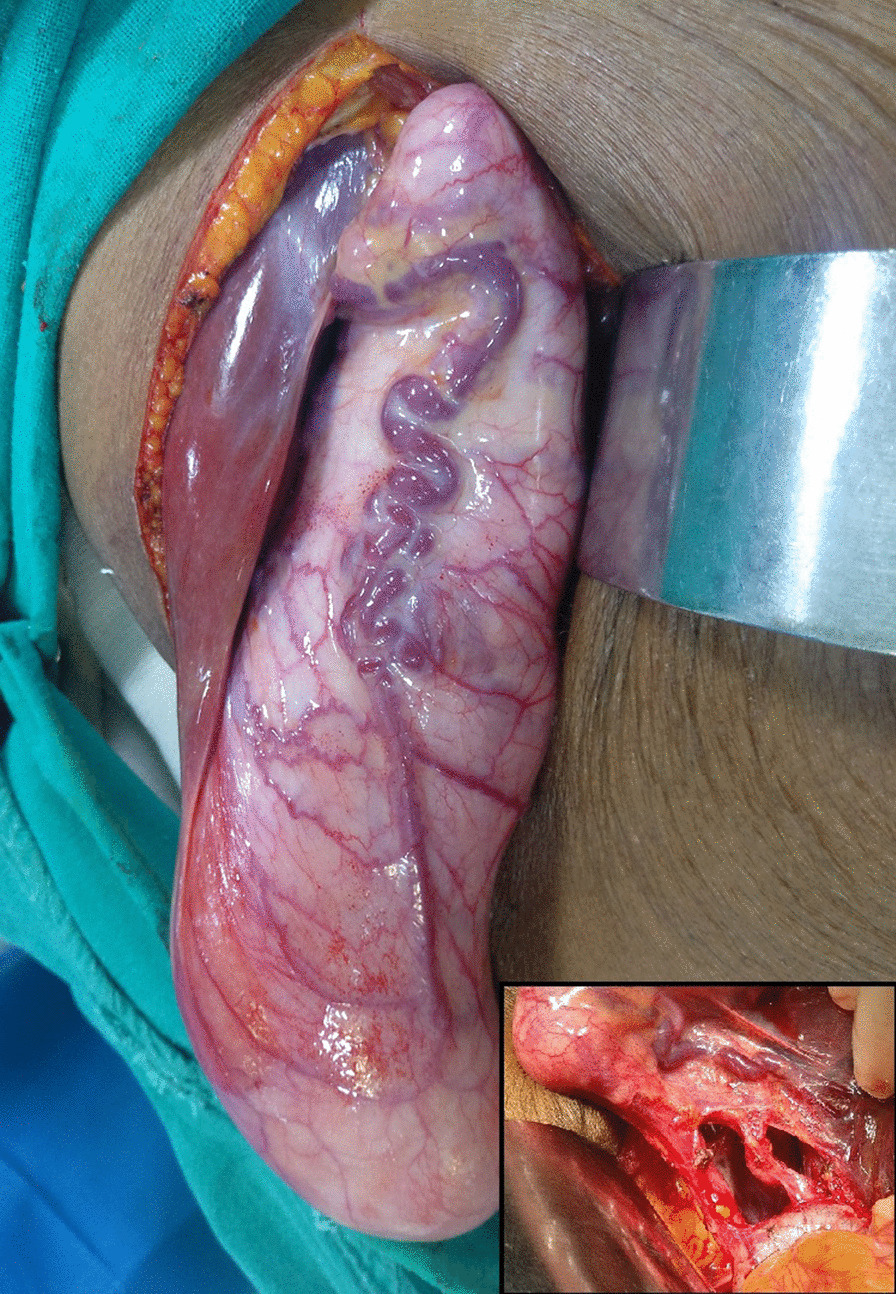
Intra-operative—24 × 9 cm GB with neovascularization over body and neck, dissected structures (inset)

**Fig. 7:**
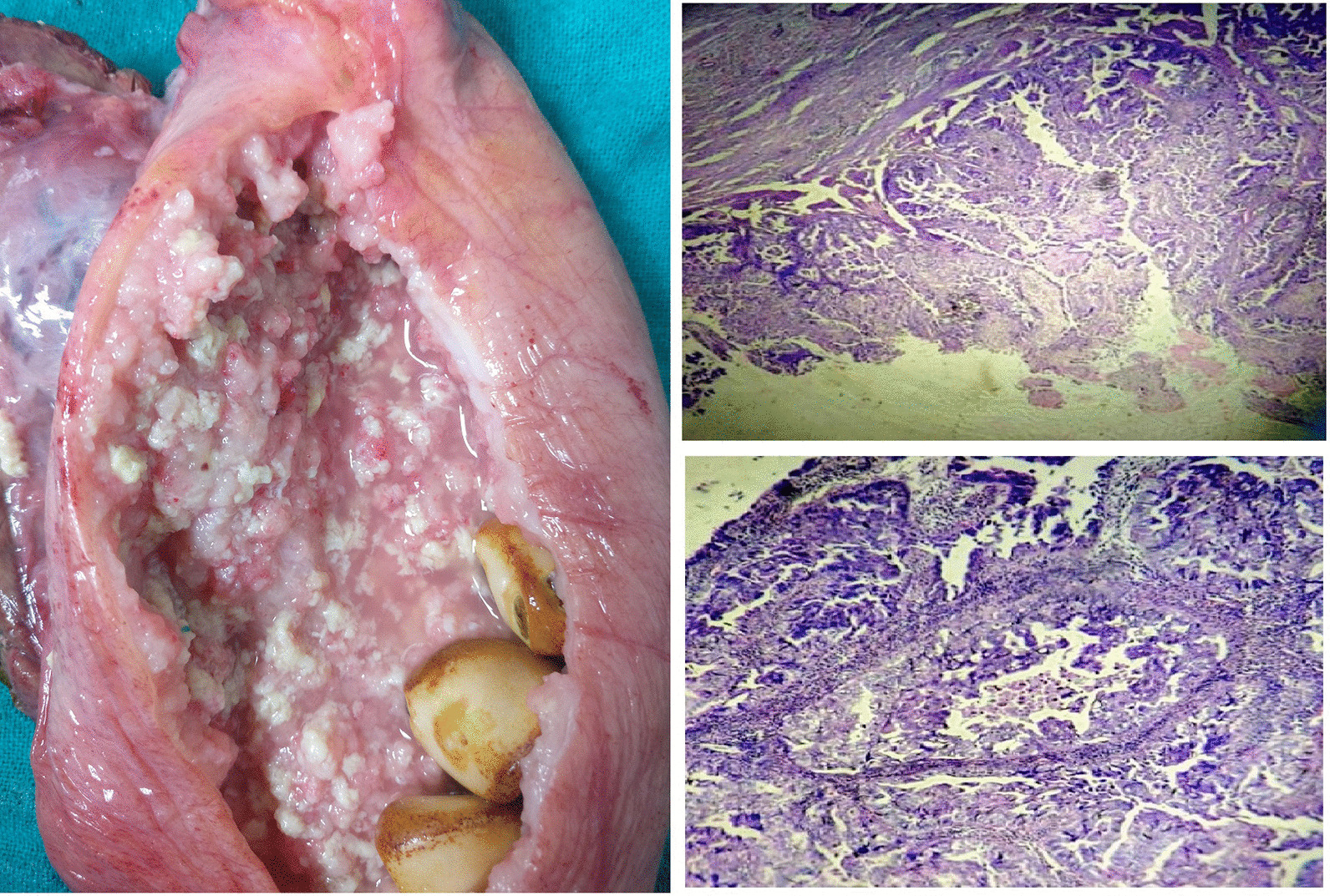
Opened specimen showing thickened irregular wall and large stones along with photomicrographs showing adenocarcinoma, H&E

As the radiological features were indicative of malignancy, patient was planned for radical cholecystectomy. At the initial diagnostic laparoscopy, no metastatic deposits seen on liver, peritoneum, omentum and no free fluid was found in the peritoneal cavity. It was followed by open radical cholecystectomy with wedge resection of liver and regional lymphadenectomy. Intra operatively a GB of size 24 × 9cm was noted (Figs. 5, 6). It was a surprise to find such a giant malignant GB with preserved planes. There were no grossly enlarged lymph nodes. The specimen was cut open and the irregularly thickened wall upto 2 cm, especially of the body and fundus was found with multiple large gall stones. Post-operative period was uneventful and patient was discharged on post-operative day 5. Histopathological examination confirmed well differentiated adenocarcinoma of gall bladder involving the muscular layer and peri-muscular tissue on the peritoneal side without serosal involvement [Stage II (T2a N0 M0)] and having a maximum wall thickness of 2.5 cm. (Fig. 7) The entire thickened wall was cancerous. Cystic duct stump margin and all other resected margins were free of tumor. All 9 lymph nodes isolated were free of tumor deposits. Patient family did not opt for further consultation with medical oncologist. Patient was healthy and disease free up to 18 months of follow up.

## Discussion and conclusion

GBC accounts for 1.2% of all global cancer diagnoses, but 1.7% of all cancer deaths [6]. According to GLOBOCAN 2018 data, GBC is the 22nd most incident but 17th most deadly cancer worldwide [6]. Women are 2 to 6 times more frequently affected as men. The incidence rate for GBC in women of North India (11.8/100,000) and North-East India (17.1/100,000) is very high compared to South and Western India (< 1/100,000 population) which is similar to the high incidence areas such as Bolivia (14/100,000), Chile (9.3/100,000) and South American (27/100,000) [2, 7]. Among the risk factors in the development of GBC, gall stones are present in 85% of patients. The relative risk (RR) of GBC with gallstone diameters of 2.0 to 2.9 cm (vs. stone size less than 1 cm) is 2.4 and for stones larger than 3 cm the risk drastically increases to 10.1. The RR of GBC increases with the duration of gall stones, with RR being 4.9 with duration of 5–19 years and RR of 6.2 for duration > 20 years [8]. The other risk factors for GB include: porcelain gall bladder, primary sclerosing cholangitis, anomalous pancreatico biliary duct junction, polyps more than 10 mm in size, solitary polyps, sessile polyps, polyps with associated gall stones, and polyps in those aged more than 65 years of age [9].

In the US, the average survival rates for Stage II, III and IV are 49, 24 and 8 months respectively, however we do not have sufficient data in India [10]. The clinical presentation of GBC is often vague or delayed relative to pathologic progression, contributing to advanced staging and dismal prognosis at the time of diagnosis [11]. Most of the patients present with vague upper abdominal pain or with an incidental finding radiologically or on histology. Incidental GBCs are detected histologically in 0.5% − 1.5% of laparoscopic cholecystectomies performed for cholelithiasis [12]. The presence of jaundice, abdominal lump, anorexia, and weight loss are usually indicative of advanced stages. A mucocele of GB in absence of any stone may be early marker for malignancy arising in cystic duct or at neck [13]. GBC are rarely diagnosed before it has advanced or metastasized [14]. GBC may arise as a nidus in pre-existing background of chronic cholecystitis, which delays the diagnosis of cancer [15]. This is evident in our case, a 65 year old female presented with non-specific symptom of abdominal pain due to gallstones for 12 years (RR-4.9) and the largest stone of size 3.5 cm (RR-10.1) with a T2a N0 M0 (Stage II) giant carcinoma gall bladder of size 24 × 9 cms.

It is important to differentiate at an early stage which eventually is likely to have better prognosis. Ultrasound (USG), computed tomography (CT), and magnetic resonance imaging (MRI) have improved the possibility of differentiating and choosing the correct treatment. Mass occupying lesion may be present in 40–65% of patients with GBC at initial detection. GBC may present as focal or diffuse asymmetric wall thickening, which can be detected by imaging techniques like contrast-enhanced CT and MRI [11]. The features suggestive of a GBC on CT are a discrete focal gallbladder mass, irregular focal wall thickening, and a ‘2-layer pattern’ of enhancement in a thickened gallbladder wall, infiltration of the surrounding structures, locoregional lymphadenopathy and metastatic deposits in the liver, peritoneum and omentum [16]. Diffuse symmetric wall thickening may imply a benign pathology, whereas asymmetric, irregular, or extensive thickening, with marked enhancement should heighten suspicion of GBC [11].

Although there are no clear-cut definitions, gallbladders of size > 14 cm and volume ≥ 1.5 L have been regarded as Giant gall bladders (GGB) [5]. There have been few articles on GGB published in literature by Panaro et al. (43 × 21 × 20 cm), Zong et al. (30 × 31 × 18 cm) and Yadav et al. (30 cm) [17–19]. Among the various GGBs only 3 such cases (including our case) were malignant (Table 1) [5, 17–25]. Chapman et al. reported a 10 × 6.5 × 0.5 cm papillary, circumferential tumor located primarily in the body and neck of the 18 cm large gallbladder and Hsu et al. reported a 16.4 × 13.6 × 7.8 cm GB with poorly differentiated adenocarcinoma [22, 24]. Junior et al. reported a case of giant squamous cell carcinoma of gall bladder infiltrating the transverse colon, however the size was not mentioned [26]. Based on the sizes mentioned in indexed literature, it appears that our case is the largest resectable GBC reported till date.

**Table 1:** Details of giant gall bladder reported till date

	Article	Age (years)	Sex	GB size (cm)	GB volume	Diagnosis
1	Panaro et al. [17]	17	NR	43 × 21 × 20	2.7 L	Byler’s disease
2	Zong et al. [18]	55	F	30 × 31 × 18	4.0 L	NA
3	Yadav et al. [19]	46	F	30	NR	Chronic cholecystitis with mucocele
4	Bains, Maranna et al., 2020 (current case)	65	F	24 × 9	–	Adenocarcinoma of gall bladder
5	Jahantab et al. [25]			22 × 6 × 1		Gangrenous cholecystitis
6	Borodach et al. [20]	67	F	20 × 12	1.5 L	NA
7	Fultang et al. [5]	63	F	19.5 × 5.4 × 5.6	NR	Chronic cholecystitis with cholelithiasis
8	Maeda et al. [21]	36	F	18 × 4	NR	Chronic cholecystitis
9	Chapman et al. [22]	59	F	18	NR	Gall bladder adenocarcinoma with liver metastasis
10	Kuznetsov et al. [23]	77	F	17.2 × 16.1 × 24.0	3.35 L	Chronic cholecystitis
11	Hsu et al. [24]	87	F	16.4 × 13.6 × 7.8	NR	Gall bladder adenocarcinoma with empyema

*NA* not available, *NR* not reported

Surgery is the mainstay of treatment of GBC which essentially is radical cholecystectomy with resection of 3 cm of liver parenchymal segments IVb and V along with regional lymphadenectomy. A minimum of 6 retrieved lymph nodes are necessary for adequate staging, indicating a thorough lymphadenectomy. Regional lymphadenectomy improves survival in T1b to T3 GBC [27]. Diagnostic Laparoscopy was performed in our patient to rule out the possibility of metastatic disease, particularly liver and peritoneal metastasis. Due to sheer size of the GB, it was decided to proceed with open resection. Staging laparoscopy avoids the need for unnecessary laparotomies in 27.6% of patients with carcinoma gall bladder [28]. Patient underwent radical cholecystectomy with albeit surprisingly no infiltration and preserved planes. In the systematic review by Gupta et al. it was found that there is an increase possibility of R0 resection by 15–86% after NACT in locally advanced (T3, T4) Ca GB [29]. 5-Flourouracil and Gemcitabine based chemotherapies have demonstrated benefit in patients with positive margins after resection, nodal positive disease and T3, T4 diseases [30]. Laparoscopic cholecystectomy for a benign GGB can be performed with adequate surgical expertise [5, 19]. Laparoscopic radical cholecystectomy for early T1 and T2 GBC has been performed in experienced centers with satisfactory results [31–33]. However open surgery is the current standard of care for malignant cases especially in giant GB.

Giant GB is an uncommon finding. They are mostly benign, however malignant cases can occur. Giant malignant GB of the size 24 × 9 cm, that too resectable is a rare finding. The features of malignancy and extent of disease must be identified in radiological scans. GBCs have a better prognosis if diagnosed and treated early as in Stage I or II. Radical cholecystectomy is the standard treatment for GBC. The staging may not correlate always with the size of gallbladder whereas prognosis depends on stage of disease and resectability, irrespective of size.

## Data Availability

Not available.

## Abbreviations

GBC: Gall bladder cancer
GGB: Giant gall bladder
GB: Gall bladder
MRCP: Magnetic resonance cholangiopancreatography
RR: Relative risk
ASIS: Anterior superior iliac spine
SEER: Surveillance, Epidemiology, and End Results
CA 19.9: Carbohydrate antigen 19.9
NACT: Neo adjuvant chemotherapy
